# Age, Food Neophobia, and Whole-Grain Acceptance in Slovenian Adolescents in the Context of Organized School Meals: Insights from the National “Whole Grain” Project

**DOI:** 10.3390/nu18060896

**Published:** 2026-03-12

**Authors:** Eva M. Čad, Anja Bolha, Blaž Ferjančič, Jasna Bertoncelj, Naja Zagorc, Mojca Korošec

**Affiliations:** Department of Food Science and Technology, Biotechnical Faculty, University of Ljubljana, 1000 Ljubljana, Slovenia; eva.marija.cad@bf.uni-lj.si (E.M.Č.); anja.bolha@bf.uni-lj.si (A.B.); blaz.ferjancic@bf.uni-lj.si (B.F.); jasna.bertoncelj@bf.uni-lj.si (J.B.); naja.zagorc@gmail.com (N.Z.)

**Keywords:** whole grain, school meals, eating habits, adolescents, acceptance

## Abstract

Background: Childhood and adolescence represent a critical period for shaping long-term dietary habits, including whole grain consumption, which remains low despite well-documented health benefits. Objective: This cross-sectional study (November–December 2024) examined Slovenian adolescents’ attitudes toward whole-grain foods in the context of organized school meals. Methods: Participants aged 10–12 years and 14–19 years (*N* = 501; mean age 15.6 ± 2.6) completed an online questionnaire assessing knowledge, self-reported consumption frequency, preferences, motivational factors, and food neophobia using the translated Italian Child Food Neophobia Scale (ICFNS). Based on ICFNS scores, participants were classified as low (≤17), medium (18–24), or high (≥25) in food neophobia. Results: Older adolescents demonstrated better knowledge of whole-grain health benefits; however, greater knowledge was not associated with higher self-reported consumption. Food neophobia was strongly associated with lower consumption frequency and reduced willingness to try whole-grain foods, including whole-grain bread, oatmeal, buckwheat porridge and brown rice. Across all groups, taste was the most consistent motivator for trying whole-grain foods. Older adolescents prioritized health and appearance as key reasons for eating more whole grain foods. Conclusions: Findings suggest that improving taste, increasing exposure, and leveraging institutional settings such as schools, where availability, preparation, and social cues can be managed, may be effective in promoting whole-grain food consumption.

## 1. Introduction

Childhood and adolescent obesity remains a public health challenge worldwide causing short- and long-term consequences for physical and mental health [1,2]. Excess weight during the early years of life is associated not only with an increased risk of chronic conditions, such as type 2 diabetes, cardiovascular disease, and certain cancers, but also with lower quality of life and psychosocial difficulties [3,4,5]. Dietary habits play a central role in the development and prevention of obesity. As such, promoting healthy food choices and dietary behaviors from a young age is a key strategy for improving population health and preventing the onset of diet-related diseases later in life.

In Slovenia, the prevalence of overweight and obesity among children and adolescents remains a growing concern, with rates of nearly 26% in boys and 23% in girls [6]. As recently reported by Poličnik [7], diets of Slovenian children and adolescents often lack key food groups such as vegetables, dairy products, nuts, seeds, legumes, and water, are low in dietary fiber, but contain excessive amounts of red meat and high-sugar foods. These findings provide valuable insight into the dietary shortcomings of Slovenian adolescents and offer a strong foundation for developing targeted public health strategies to improve their eating habits.

Schools play a key role in shaping children’s eating habits due to their established meal provision infrastructure. In Slovenia, school meals are provided through a legally regulated system. In primary schools, meals are usually prepared in school canteens and planned by designated food service managers, whereas secondary schools mostly receive meals from distribution kitchens run by external providers. Children typically consume one to four meals per day at school, providing between 20% and 50% of their total energy intake, or even more if breakfast and afternoon snacks are included [7]. As part of this framework, schools must follow the National Guidelines for Nutrition in Educational Institutions [8], which promote the inclusion of whole grain ingredients and specify their recommended frequency in school meals. The organized school meal system, together with curriculum-based food education, therefore provides structured opportunities for controlled exposure and gradual menu reformulation. In addition, the shared mealtime environment facilitates social norm formation, as peer and staff influences may normalize the regular inclusion of whole-grain foods [9]. Hence, schools offer a valuable opportunity to promote healthy eating habits during a critical stage of development and can serve as consistent source of balanced nutrition, especially to children from socioeconomically disadvantaged backgrounds [7,10].

Evidence from recent systematic reviews suggests that school-based nutrition interventions can have a positive effect on dietary outcomes [11,12]. One area of focus within these interventions is the improvement of food quality, particularly through the inclusion of nutrient-rich options such as whole-grain foods. As part of these efforts, the inclusion of whole-grain foods in school meals presents both nutritional and sustainability benefits. In this study, whole-grain foods were defined as products containing all parts of the grain kernel, including the bran and germ, whether intact or milled into smaller pieces [13]. Whole-grain foods, which are higher in dietary fiber and micronutrients compared to their refined counterparts, are associated with improved long-term health outcomes and offer environmental benefits due to their lower degree of processing [14,15,16]. Despite these advantages, whole-grain foods are frequently rejected by children and adolescents, often due to their darker color, coarser texture, or unfamiliar taste [17,18]. This reluctance is especially pronounced among youth with higher levels of food neophobia [19].

Food neophobia refers to the reluctance or refusal to eat unfamiliar foods and is considered a common trait among children and adolescents [20,21,22,23]. It is derived from the earlier work of Rozin’s “omnivore’s dilemma” [24]. While it may serve an evolutionary protective function, high levels of food neophobia can pose challenges for both public health and nutrition education [22]. Recent validation of the Child Food Neophobia Scale in Italy has proven it to be a reliable tool to assess this trait in younger populations [25]. Numerous studies have demonstrated a negative association between food neophobia and dietary variety, with neophobic children typically consuming fewer fruits, vegetables, and whole-grain products [19,22,23,26]. However, most of this evidence focuses on overall dietary diversity or fruit and vegetable intake, whereas grain-specific acceptance, particularly of staple products such as bread, pasta, and porridge, has been studied less, especially in the context of organized school meal systems. Given the nutritional and environmental benefits of whole grains, it is crucial to better understand the specific motivators that could encourage adolescents, especially those with higher food neophobia, to try and accept whole-grain foods in a school environment.

Food choices among children and adolescents are shaped by a combination of factors, including personal preferences, peer influence, family habits, knowledge, food availability, and media exposure [27]. Among these, taste and familiarity play a central role in driving long-term consumption [28]. To support healthier dietary patterns among Slovenian youth, particularly in increasing the intake of whole-grain foods in a school environment, it is essential to understand the psychological and behavioral factors that shape their choices. Although previous research has examined whole grain intake and food neophobia separately, few studies have jointly investigated age and food neophobia as drivers of whole grain acceptance within the structured context of organized school meals, particularly among Slovenian adolescents. This study therefore aimed to explore the consumption frequency, attitudes, and motivations of Slovenian adolescents regarding whole grain consumption in the context of organized school meals, with a focus on the role of food neophobia and age. This work was conducted as part of the national targeted research project “Whole Grain” (V3-2310), aiming to strengthen the link between nutrition education and organized school meals.

## 2. Materials and Methods

### 2.1. Project and Study Design

As part of the “Whole Grain” (V3-2310) project, which uses whole-grain foods as a model for promoting healthier dietary habits among children and adolescents and improving the link between nutrition education and organized school meals, the present study investigated adolescents’ attitudes, preferences, and perceptions toward products containing whole-grain ingredients. The survey instrument was developed and validated within the project and approved by the Ethical Committee at the National Institute of Public Health of Slovenia (approval no. 631-6/2024-14 (013)). The sample size was determined pragmatically based on the number of participating schools and classes that agreed to take part in the survey during the study period, as the survey represented one component of the broader project.

Primary and secondary schools in Ljubljana and the surrounding areas, Slovenia’s most densely populated areas, were invited through existing collaborations and outreach to teachers interested in food- and nutrition-related projects. Within participating schools, teachers informed eligible classes about the study and coordinated survey administration during school hours, when they decided to participate.

### 2.2. Participants

A total of 501 adolescents participated. Two age groups were targeted: 10–12 years (5th–6th grade of primary school) and those aged 14 years and older (all four grades of secondary school or gymnasium). These age categories were selected to reflect the structural transition from primary to secondary school within the Slovenian education system, where differences in school meal provision, daily schedule, and adolescents’ autonomy in food selection may influence eating behavior. Participation was voluntary and anonymous, and written parental/guardian informed consent was obtained for all minors. The study complied with the General Data Protection Regulation (GDPR); no personal identifiers or sensitive data were collected, and responses cannot be traced back to individuals.

### 2.3. Questionnaire

The questionnaire (File S1, Supplementary Materials), which was developed within the national research project “Whole Grain” (V3-2310), examined adolescents’ knowledge, consumption frequency, attitudes and preferences towards whole grains, as well as their reluctance to eat unfamiliar foods (neophobia). Whole-grain foods included in the questionnaire reflect grain-based products commonly consumed in Slovenia and frequently offered within the organized school meal system.

The questionnaire consisted of four domains: (1) knowledge of whole-grain foods (image identification and true/false statements), (2) reported consumption frequency of grain-based foods (4-point scale), (3) attitudes and preferences related to whole-grain foods, including motivators and preparation preferences (4- and 5-point scales), and (4) food neophobia assessed using the 8-item Italian Child Food Neophobia Scale (ICFNS; 5-point scale).

Food neophobia was measured using the translated Italian Child Food Neophobia Scale (ICFNS) [25], which was translated into Slovene and checked for clarity by two researchers. Following the procedure of Laureati et al. (2015) [25], participants were divided into three food neophobia groups based on their ICFNS scores: low neophobia (scores ≤ 17; *n* = 106) medium neophobia (scores = 18–24; *n* = 236), and high neophobia (scores ≥ 25; *n* = 165). The internal consistency of the Child Food Neophobia Scale was satisfactory (Cronbach’s α = 0.67).

The whole questionnaire, including ICFNS, was pilot tested for clarity with a group of five pupils from the 5th and 6th grades. The overall structure and terminology of the questionnaire were adapted to ensure age-appropriateness and comprehension among both primary- and secondary-school participants. Questions were designed to be clear, concise, and engaging for younger respondents, incorporating food selection and preparation methods characteristic of study-specific context. To assess response reliability and consistency, the statement “Whole grain pasta usually takes longer to cook than pasta made from white flour” was included twice in the questionnaire. Agreement between the two responses indicated substantial consistency (Cohen’s κ = 0.68). A Chi-square test further confirmed a significant association between the two responses (χ^2^(1) = 229.8, *p* < 0.001), suggesting that participants answered consistently and thoughtfully throughout the questionnaire.

The questionnaire was implemented and distributed via an online platform (1KA, version 24.06.14, www.1ka.si), and the survey link was sent by e-mail to participating schools. Students completed the questionnaire during school hours, either individually or in supervised classroom settings, depending on the school’s internal arrangements. Data collection took place between 12 November and 20 December 2024.

### 2.4. Data Analysis

All analyses were conducted using R (version 4.4.0). Descriptive statistics were used to summarize participant characteristics. Reliability of the Child Food Neophobia Scale was assessed by calculating internal consistency (Cronbach’s alpha). To examine differences in responses between age groups (10–12 vs. 14–19 years) and food neophobia levels (low, medium, high), categorical data and responses to questions were analyzed using either Pearson’s Chi-square test or Fisher’s exact test, depending on whether assumptions regarding expected cell counts were met. Fisher’s exact test was used in cases where expected counts were below 5 in more than 20% of cells. When Pearson’s Chi-square or Fisher’s exact test revealed a statistically significant difference across food neophobia groups, pairwise post hoc comparisons were conducted to identify which groups differed, using Bonferroni correction to adjust for multiple testing. Analyses focused on bivariate group comparisons; no multivariable adjustment was performed. A significance level of *p* < 0.05 was used for all analyses.

## 3. Results

### 3.1. Participant Characteristics

A total of 501 children and adolescents participated in the study, including 88 in the 10–12 and 413 in the 14–19 age group (Table 1). The groups differed significantly in age, gender distribution, food neophobia levels, and knowledge of whole-grain foods (all *p* < 0.05). The older group included more females and had a higher proportion of participants with high food neophobia, while knowledge of whole grains was also higher among older participants.

### 3.2. Knowledge of Whole-Grain Foods

Knowledge of whole-grain foods was assessed through image identification (Q10, Supplementary Materials) and true/false statements about general characteristics of whole-grain foods (Q11, Supplementary Materials), whole-grain bread (Q12, Supplementary Materials), and whole-grain pasta (Q13, Supplementary Materials). Results were compared between younger (10–12) and older (14–19) adolescents using Chi-squared tests (Tables S1–S4, Supplementary Materials).

#### 3.2.1. Identification of Whole Grain Foods

Ability to correctly identify whole-grain foods from photographs varied across food types. Recognition was highest for whole-grain bread (96.8%), whole-grain rice (95.5%), and whole-grain pasta (95.1%), with no significant differences between age groups. However, only 11.0% of participants correctly identified popcorn as a whole-grain food, regardless of age (χ^2^(1) = 0.02, *p* = 0.90). The older age group was significantly more accurate in identifying light bread with seeds as not whole grain (37.8% vs. 21.3%; χ^2^(1) = 8.0, *p* = 0.005) and recognizing millet groats as a whole-grain food (53.9% vs. 34.4%; χ^2^(1) = 11.0, *p* = 0.001). No other food items showed statistically significant differences between age groups (Table S1, Supplementary Materials).

#### 3.2.2. General Knowledge About Whole-Grain Foods

Most participants correctly responded that consuming whole-grain foods is healthy (90.7%) and that whole-grain flour is recommended to be stored in the refrigerator (78.5%). The older age group was significantly more likely to correctly identify the structural parts of a whole grain (χ^2^(1) = 39.6, 72.5% vs. 37.1%; *p* < 0.001) and to know that whole-grain pasta usually requires longer cooking time than refined pasta (χ^2^(1) = 12.5, 55.0% vs. 33.7%; *p* < 0.001). No other statements in this section showed significant differences between the two age groups (Table S2, Supplementary Materials).

#### 3.2.3. Knowledge About Whole-Grain Bread

While most participants understood that whole-grain bread contains ground grain kernel (63.3%) and is darker in color (87.4%), several misconceptions were observed. The older age group was significantly more likely to reject the incorrect statement that “any bread topped with seeds is whole grain” (83.0% vs. 68.5%; χ^2^(1) = 8.9, *p* = 0.003) and to correctly recognize its higher vitamin and mineral content (82.1% vs. 68.5%; χ^2^(1) = 7.5, *p* = 0.006). Interestingly, the younger group was more likely to believe that whole grain-bread stays fresh longer than white bread (56.2% vs. 44.3%; χ^2^(1) = 3.7, *p* = 0.05).

#### 3.2.4. Knowledge About Whole-Grain Pasta

Most participants correctly stated that whole-grain pasta is not the same color as white pasta (88.4%) and that it can be more satiating (64.1%). The older age group was significantly more accurate in recognizing that the two types differ in color (90.2% vs. 79.8%; χ^2^(1) = 6.8, *p* = 0.009) and in knowing that whole-grain pasta generally takes longer to cook (51.7% vs. 38.2%; χ^2^(1) = 4.8, *p* = 0.03). No significant differences were found in other statements related to labeling or satiety (Table S4, Supplementary Materials).

### 3.3. Consumption Frequency of Whole-Grain Foods

**Figure 1:**
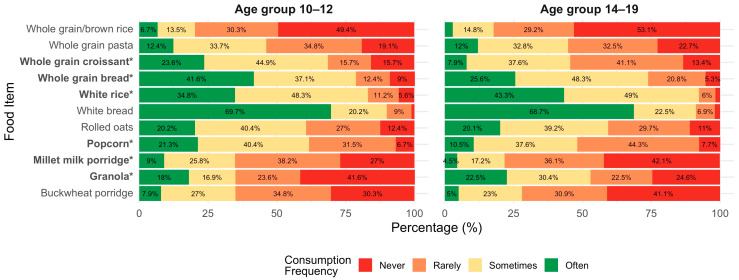
Frequency of grain-based food consumption by age group (10–12 vs. 14–19 years). Stacked bar chart showing the percentage distribution of responses across four consumption frequency categories (never, rarely, sometimes, often). Statistical differences between age groups were assessed using Fisher’s exact test. Food items marked with * differ significantly between groups (*p* < 0.05). Percentages below 4% are not displayed to improve readability; displayed values may not sum exactly to 100% due to rounding.

Consumption frequency of whole-grain foods was assessed by asking participants how frequently they consumed specific foods, using a 4-point scale with responding options: “never”, “rarely”, “sometimes” and “often” (Q14, Supplementary Materials). Responses were compared across age groups and food neophobia levels. Figure 1 presents the frequency of consumption of various grain-based (refined and whole grain) foods among participants in the two age groups (10–12 years and 14–19 years). Across both groups, white bread and white rice were among the most frequently consumed items. Several age-related differences emerged in the consumption of whole-grain foods. Compared to the younger group, participants aged 14–19 reported significantly lower consumption of whole-grain croissants (*p* < 0.001), whole-grain bread (*p* = 0.005), millet milk porridge (*p* = 0.016), and popcorn (*p* = 0.023), but consumed granola more frequently (*p* = 0.005). White rice consumption also frequency differed significantly between age groups (*p* = 0.031), with younger adolescents consuming it more frequently. In contrast, no significant age-related differences were found in the consumption of buckwheat porridge (*p* = 0.221), white bread (*p* = 0.839), whole-grain pasta (*p* = 0.900), brown rice (*p* = 0.336), or rolled oats (*p* = 0.946).

**Figure 2:**
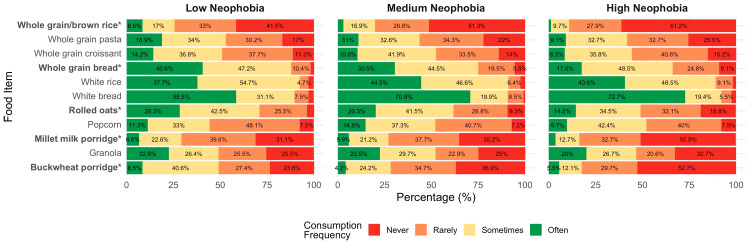
Frequency of grain-based food consumption by neophobia level (low vs. medium vs. high). Stacked bar chart showing the percentage distribution of responses across four consumption frequency categories (never, rarely, sometimes, often). Statistical differences between neophobia groups were assessed using Fisher’s exact test. Food items marked with * differ significantly between groups (*p* < 0.05). Percentages below 4% are not displayed to improve readability; displayed values may not sum exactly to 100% due to rounding.

Figure 2 displays the reported frequency of consumption of whole-grain and refined foods across participants categorized by food neophobia level (low, medium, high). Participants with high levels of food neophobia reported significantly lower consumption of several whole-grain foods compared to those with lower neophobia. These included whole-grain bread (*p* = 0.001), rolled oats (*p* = 0.003), buckwheat porridge (*p* < 0.001), millet milk porridge (*p* = 0.018), and whole-grain/brown rice (*p* = 0.003). Post hoc comparisons showed that these differences were observed between the high- and low-neophobia groups, with participants in the high-neophobia group significantly more likely to report “never” or “rarely” consuming these foods (adjusted *p* < 0.05 for all comparisons). In contrast, no significant differences were found across neophobia levels for white bread, white rice, granola, popcorn, and whole-grain croissants (*p* > 0.05 for all).

### 3.4. Attitudes Towards Whole-Grain Foods

#### 3.4.1. Factors Influencing Whole-Grain Food Intake

Participants were asked which factors would encourage them to eat more whole-grain foods (Q15, Supplementary Materials). Overall, the most chosen factors that would encourage students to eat more whole-grain foods were the belief that whole grains are good for their health, the potential for whole grains to help achieve a nicer body shape, and more frequent consumption of whole grain foods at home.

**Figure 3:**
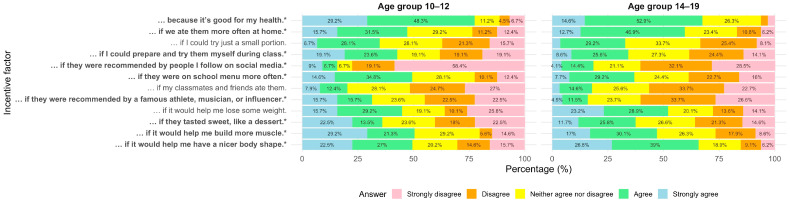
Self-reported factors for increasing whole-grain consumption by age group (10–12 vs. 14–19 years). Stacked horizontal bar chart showing the percentage distribution of responses across five agreement categories (strongly disagree to strongly agree) to a statement: »I would eat whole grain foods more often …«. Statistical differences between age groups were assessed using Fisher’s exact test. Statements marked with * differ significantly between groups (*p* < 0.05). Percentages below 4% are not displayed to improve readability; displayed values may not sum exactly to 100% due to rounding.

**Figure 4:**
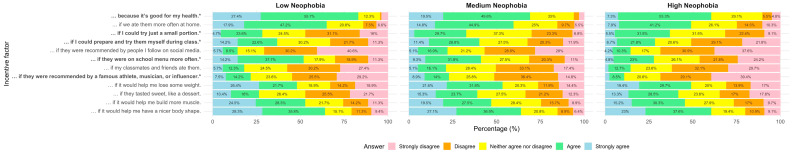
Self-reported factors for increasing whole-grain consumption by neophobia level (low vs. medium vs. high). Stacked horizontal bar chart showing the percentage distribution of responses across five agreement categories (strongly disagree to strongly agree) to a statement: »I would eat whole grain foods more often …«. Statistical differences between neophobia groups were assessed using Fisher’s exact test. Statements marked with * differ significantly between groups (*p* < 0.05). Percentages below 4% are not displayed to improve readability; displayed values may not sum exactly to 100% due to rounding.

Age group and food neophobia level significantly influenced responses to several attitudinal statements (Figure 3 and Figure 4, Table S5, Supplementary Materials). For example, participants aged 10–12 were significantly more likely than those aged 14–19 to report being encouraged by the health benefits of whole grains (*p* < 0.001), being able to prepare and try them during class (*p* = 0.026), if they were recommended by celebrities (*p* = 0.003), and if they were available more frequently in the school cafeteria (*p* = 0.017). In contrast, older adolescents were more likely to be influenced by factors related to eating them more often at home (*p* = 0.047), social media (*p* < 0.001), sweet taste (*p* = 0.007), and improved appearance or physique (*p* < 0.01).

Significant differences between food neophobia groups were found for several statements describing conditions under which participants would be more willing to eat whole-grain foods (Table S5, Supplementary Materials). Overall Fisher’s exact tests indicated significant effects of food neophobia for the following statements: “because it’s good for my health” (*p* = 0.001), “if I could try just a small portion” (*p* = 0.051), “if I could prepare and try them myself during class” (*p* = 0.014), “if they were on the school menu more often” (*p* = 0.001), and “if they were recommended by a famous athlete, musician, or influencer” (*p* = 0.001). Participants with high food neophobia were significantly less likely than those with low or moderate neophobia to report being encouraged by the health benefits of whole grains (*p* < 0.001 vs. low; *p* = 0.020 vs. middle), by their availability on the school menu (*p* = 0.002 vs. low; *p* = 0.011 vs. middle), and by the opportunity to prepare and try them during class (*p* = 0.014 vs. middle). Additionally, participants with low food neophobia were more likely than those with moderate neophobia to be encouraged by the option to try a small portion (*p* = 0.027). No significant differences between food neophobia groups were observed for the remaining statements.

#### 3.4.2. Preferences for Including Grain-Based Foods in School Meals

**Figure 5:**
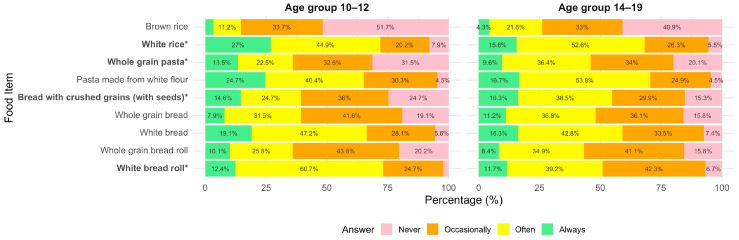
Self-reported preferences for including grain-based foods in school snacks or lunches by age group (10–12 vs. 14–19 years). Stacked bar chart showing the percentage distribution of responses across four preference categories (never to always). Statistical differences between age groups were assessed using Fisher’s exact test. Food items marked with * differ significantly between groups (*p* < 0.05). Percentages below 4% are not displayed to improve readability; displayed values may not sum exactly to 100% due to rounding.

**Figure 6:**
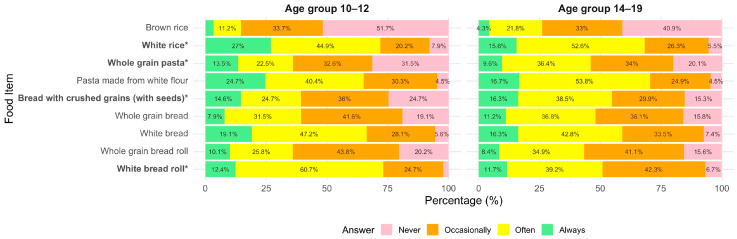
Self-reported preferences for including grain-based foods in school snacks or lunches by neophobia level (low vs. medium vs. high). Stacked bar chart showing the percentage distribution of responses across four preference categories (never to always). Statistical differences between neophobia groups were assessed using Fisher’s exact test. Food items marked with * differ significantly between groups (*p* < 0.05). Percentages below 4% are not displayed to improve readability; displayed values may not sum exactly to 100% due to rounding.

Participants were asked how often they would like specific grain-based foods to be included in school snacks or lunches (Q16, Supplementary Materials). As shown in Figure 5 and Figure 6, whole-grain bread and pasta were the most preferred options among the whole-grain foods. Some differences emerged based on both age group and food neophobia level. Younger adolescents (10–12 years) were significantly more likely than older adolescents (14–19 years) to prefer the inclusion of whole-grain pasta (*p* = 0.020) and bread with crushed grains and seeds (*p* = 0.032). Preferences for other whole-grain items, including whole-grain bread and brown rice, did not differ significantly between age groups (all *p* > 0.05, Table S6, Supplementary Materials).

Participants with high food neophobia were significantly less likely to prefer the inclusion of whole-grain bread (*p* < 0.001), brown rice (*p* = 0.021), and bread with seeds (*p* = 0.001) in school meals. Post hoc tests indicated that these differences were significant between the high- and low-neophobia groups for whole-grain bread (adjusted *p* < 0.001), brown rice (adjusted *p* = 0.036), and bread with seeds (adjusted *p* < 0.01), as well as between the medium- and low-neophobia groups for whole-grain bread (adjusted *p* < 0.001) and bread with seeds (*p* = 0.030). No significant differences by neophobia level were observed for white bread, white rice, white bread rolls, or whole-grain pasta.

#### 3.4.3. Preferred Preparation Method for Oatmeal

A question about oatmeal preparation was included in the questionnaire because oatmeal is a commonly eaten food for breakfast or as a snack, both at school and at home in Slovenia (Q17, Supplementary Materials). Adolescents, regardless of age, were most inclined to taste and eat oatmeal at school when it was prepared “with milk”, “with milk and cocoa powder”, or “with chocolate pieces, milk, and dried fruit” (Figures S1 and S2, Supplementary Materials). The least preferred option across both age groups was oatmeal prepared with a plant-based drink (e.g., soy, oat, or almond drink). Statistically significant age-related differences were observed only for the option “with fruit yogurt” (*p* = 0.024), with younger participants (10–12 years) being less likely to choose this option. For all other preparation methods, including those with plant-based drinks, dairy, nuts, dried fruit, or chocolate, no significant age-based differences were found (*p* > 0.05, Table S7, Supplementary Materials).

Preferences for how oatmeal should be prepared at school varied significantly across food neophobia groups (Table S7, Supplementary Materials). Fisher’s exact tests indicated significant group differences for oatmeal prepared with milk (*p* < 0.001), plain yogurt (*p* = 0.001), milk and dried fruit (*p* = 0.002), milk and nuts (*p* = 0.002), fruit yogurt (*p* = 0.031), and chocolate pieces, milk, and dried fruit (*p* = 0.006). Post hoc comparisons revealed that participants with high food neophobia were significantly less likely to prefer oatmeal prepared with milk (*p* < 0.001 vs. low; *p* = 0.016 vs. middle), plain yogurt (*p* = 0.001 vs. low; *p* = 0.008 vs. middle), milk and dried fruit (*p* < 0.001 vs. low; *p* = 0.009 vs. middle), milk and nuts (*p* < 0.001 vs. low; *p* = 0.033 vs. middle), and chocolate pieces, milk, and dried fruit (*p* = 0.012 vs. low; *p* = 0.018 vs. middle). Additionally, high-neophobia participants were less likely than those with moderate neophobia to prefer oatmeal prepared with fruit yogurt (*p* = 0.021). No significant differences between food neophobia groups were observed for oatmeal prepared with milk and cocoa powder or with a plant-based drink (soy, oat, almond).

#### 3.4.4. Motivations for Trying New Foods

Participants were asked what would most encourage them to try a dish they had never eaten before (Q19, Supplementary Materials). Overall, the biggest motivator for trying new foods was the good taste of the foods on the first try.

**Figure 7:**
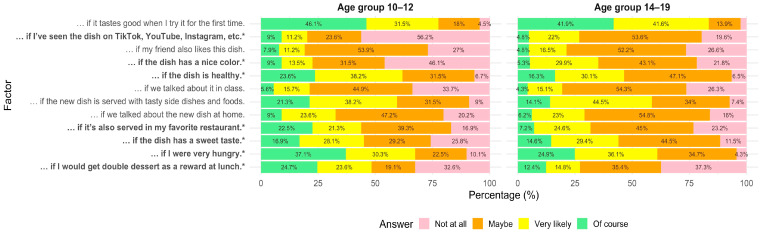
Motivators for trying new foods by age group (10–12 vs. 14–19 years). Stacked bar chart showing the percentage distribution of responses across four likelihood categories (not at all to of course) to a statement: I’ll try an unfamiliar dish and start eating it regularly if …«. Statistical differences between age groups were assessed using Fisher’s exact test. Statements marked with * differ significantly between groups (*p* < 0.05). Percentages below 4% are not displayed to improve readability; displayed values may not sum exactly to 100% due to rounding.

As shown in Figure 7, several motivators differed between age groups. Younger participants (10–12 years) were significantly more likely than older participants (14–19 years) to be encouraged by external cues such as receiving a reward (dessert) (*p* < 0.001), being very hungry (*p* = 0.006), visual appeal of the dish (*p* < 0.001), a sweet taste (*p* = 0.003), and familiarity via social media platforms (*p* < 0.001). The younger group also showed greater influence from the dish being served in a favorite restaurant (*p* < 0.001). No other age-related differences in responses to intrinsic or social motivators were observed (*p* > 0.05, Table S8, Supplementary Materials).

**Figure 8:**
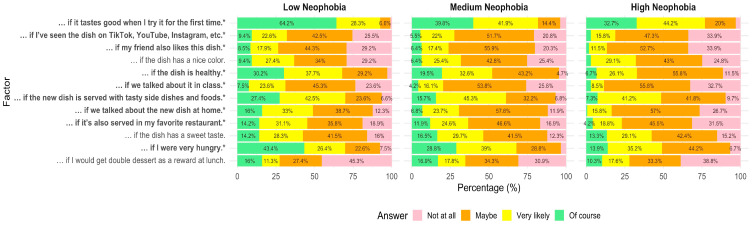
Motivators for trying new foods by neophobia level (low vs. medium vs. high). Stacked bar chart showing the percentage distribution of responses across four likelihood categories (not at all to of course) to a statement: I’ll try an unfamiliar dish and start eating it regularly if …«. Statistical differences between neophobia groups were assessed using Fisher’s exact test. Statements marked with * differ significantly between groups (*p* < 0.05). Percentages below 4% are not displayed to improve readability; displayed values may not sum exactly to 100% due to rounding.

Furthermore, several motivational factors differed between low-, medium-, and high-neophobia groups (Figure 8). Participants with high food neophobia were consistently less likely to be motivated by being very hungry (*p* < 0.001), the dish being healthy (*p* < 0.001), tasting good on first try (*p* < 0.001), served with tasty side dishes (*p* < 0.001), being served in a favorite restaurant (*p* = 0.001), and peer or social endorsement, such as friends liking the dish (*p* = 0.003) or having discussed the dish at home (*p* < 0.001). Post hoc comparisons revealed that these differences were particularly pronounced between the high- and low-neophobia groups (adjusted *p*-values < 0.001 for items such as “… if I were very hungry,” “… if the dish is healthy,” and “… if we talked about the new dish at home”). Medium-neophobia participants generally fell between the two extremes but were still significantly different from the high-neophobia group on several items (detailed results presented in Table S9, Supplementary Materials).

## 4. Discussion

This study examined adolescents’ knowledge, consumption patterns, preferences, and motivational drivers related to whole-grain foods, with a specific focus on the impact of age and food neophobia. The findings offer important insights into behavioral and psychological factors that influence whole grain acceptance among Slovenian youth and identify several points for school-based nutrition interventions.

Generally, adolescents in this study demonstrated good knowledge of whole-grain foods, particularly regarding health benefits. Interestingly, only a small proportion of participants correctly identified popcorn as a whole-grain food. In the Slovenian context, popcorn is more commonly perceived as a snack food rather than categorized within grain-based staple foods, and it is rarely framed explicitly as a “whole-grain” product in everyday communication. Therefore, the low recognition rate may reflect differences in cultural categorization or product framing rather than a lack of knowledge about whole grains.

Older adolescents showed more knowledge, particularly in distinguishing between whole-grain and refined products and in understanding that whole grains contain more vitamins and minerals. This is probably due to the natural progression in cognitive development and food literacy with age [29,30]. However, despite greater knowledge, older adolescents reported lower consumption frequency of several whole-grain foods (e.g., millet porridge, whole-grain croissants) compared to the younger group (10–12), suggesting that knowledge alone does not predict intake behavior. This discrepancy reflects the well-documented knowledge–behavior gap in nutrition research. According to the Capability, Opportunity, Motivation-Behavior (COM-B) model [31], behavior occurs when individuals have sufficient capability, opportunity, and motivation [31,32]. While older adolescents demonstrated greater knowledge (psychological capability), this alone was not associated with higher consumption, suggesting that motivational factors and environmental opportunities may play a more decisive role in shaping whole grain intake. Discrepancy in consumption frequency between age groups may also be partially explained by differences in food autonomy: younger adolescents’ diets are still largely influenced by parental choices, while older adolescents have more independence and make more of their own food decisions, often purchasing food themselves [33,34]. This discrepancy aligns with research suggesting that adolescents, as they gain independence, may prioritize taste, convenience, or peer influence over health considerations [35].

Food neophobia was associated with lower whole grain consumption frequency, reduced preference for whole grain inclusion in school meals, and decreased willingness to try whole-grain foods. These findings are consistent with previous research showing that food neophobia is associated with reduced dietary variety and lower intake of nutrient-dense foods such as vegetables, legumes, and whole grains [22,23,26,36,37]. Notably, participants with high neophobia levels were less likely to consume common whole grain staples like whole-grain bread and oatmeal. These findings highlight that food neophobia may be an important barrier to the acceptance of whole grain-rich foods and should be considered in efforts to improve adolescent dietary quality.

In general, the most influential factors encouraging adolescents to eat more whole-grain foods were the belief that whole grains are good for their health, the perception that they may help achieve a nicer body shape, and more frequent consumption of whole-grain foods at home. These findings suggest that both internal motivations (such as health and body image) and environmental influences (like family habits) play an important role in shaping participants’ dietary intake. In addition, younger and less neophobic participants were more responsive to experiential motivators, such as opportunities to prepare and try whole-grain foods during class and increased availability in school menus. These findings highlight the opportunity for schools to act as controlled environments where healthy dietary habits can be encouraged through hands-on activities and provision. Provision of whole-grain foods in the school environment would not only improve availability but also facilitate exposure, an important factor in increasing familiarity and acceptance [38,39]. Over time, repeated exposure may enhance liking, even among neophobic individuals, who often require more encounters with unfamiliar foods before accepting them [40].

Importantly, for all participants, including those with high food neophobia, the most consistent motivator for trying whole-grain foods was simply that they “taste good,” underscoring the importance of offering appealing, well-prepared whole grain options. This emphasis on taste aligns with previous research showing that taste is a primary driver of food choice [41,42], even outweighing nutritional considerations [43]. How food is prepared, specifically oatmeal, can also play a significant role in acceptance, as preparation methods directly influence taste. For example, oatmeal made with regular milk or chocolate was preferred over versions prepared with plant-based drinks or fruit yogurt, especially among high-neophobia participants. This highlights that small modifications in how whole grains are prepared and presented may help increase acceptance among reluctant eaters. Furthermore, younger adolescents responded more strongly to sensory appeal, rewards, and peer/social cues. In contrast, adolescents placed more importance on aesthetic, health, and social media-related motivators. Participants with high food neophobia, however, were consistently less responsive to all motivators, except for taste. These results imply that interventions should be tailored but ultimately suggest that if whole-grain foods taste good, youth, regardless of age or food neophobia, are more likely to accept and consume them. In practical terms, this may include gradual reformulation of grain products to increase whole-grain content over time, alongside optimizing preparation methods in school kitchens through targeted staff training to enhance sensory quality and palatability.

This study provides novel insights into whole grain acceptance among Slovenian adolescents and is among the first to explore food neophobia in this population using the translated Child Food Neophobia Scale. Although the Child Food Neophobia Scale has not yet been formally validated in Slovenian adolescents, its internal reliability in our sample was acceptable (Cronbach’s α = 0.67), and the observed associations between neophobia, preferences, and consumption frequency align with other findings [25,44,45,46]. Although internal consistency was acceptable, formal validation of the translated scale, including factor structure and test–retest reliability, is needed in Slovenian adolescents. The prevalence of high food neophobia in our sample (33%) was also consistent with rates reported in studies conducted in the US (32.8%, Falciglia et al. (2000) [47]; Galloway et al. (2003) [36]), UK (27.1%, Maratos & Staples (2015) [48]), Spain (28.7%, Maiz & Balluerka (2018) [49]), and Finland (24.5%, Kähkönen et al. (2018) [50]). As data were self-reported and collected in school settings, some response bias may be present. Furthermore, we did not collect data on socioeconomic status or parental education, which may influence food neophobia and dietary behavior. Although some demographic variables were available, residual confounding cannot be excluded. In addition, because schools were recruited through existing collaborations in Ljubljana and surrounding areas, the sample cannot be considered nationally representative. Schools with greater interest in nutrition-related topics may be overrepresented, potentially influencing knowledge and attitude estimates. Nevertheless, this study contributes valuable data to the growing body of research on adolescent dietary behavior. Given the high bread intake in Slovenia, averaging 210 g/day (men) and 144 g/day (women) among adults [51] and 164 g/day (boys) and 119 g/day (girls) among adolescents [7], and the presence of centrally regulated school meals, these findings highlight opportunities to reformulate staple products and utilize institutional settings to promote whole grain consumption.

## 5. Conclusions

While our study did not directly assess the effects of school meal composition, our findings align with the existing literature, suggesting that increasing the availability of whole grains in school settings may support healthier eating behaviors. The observed associations between preferences, age, and food neophobia highlight the potential value of combining knowledge-building, exposure, and tailored environmental availability as a strategy to encourage whole grain consumption. The school environment thus remains a key setting for shaping food preferences, reducing neophobia, and normalizing whole grain consumption.

## Figures and Tables

**Table 1:** Participant characteristics by age group (*N* = 501).

Characteristic	Total (*N* = 501)	Age Group 10–12 (*n* = 88)	Age Group 14–19 (*n* = 413)	*p*-Value
Gender				
Male	360 (71.9%)	45 (51.1%)	315 (76.3%)	<0.001
Female	135 (26.9%)	42 (47.7%)	93 (22.5%)	
Other	6 (1.2%)	1 (1.1%)	5 (1.2%)	
Age (mean ± SD)	15.6 ± 2.6	10.6 ± 0.6	16.6 ± 1.2	<0.001
Food Neophobia Level				
Low	104 (20.8%)	22 (25.0%)	82 (19.8%)	0.04
Medium	234 (46.7%)	47 (53.4%)	187 (45.3%)	
High	163 (32.5%)	19 (21.6%)	144 (34.9%)	
Heard of Whole Grain foods?				
Yes	472 (94.2%)	74 (84.1%)	398 (96.4%)	<0.001
No	29 (5.8%)	14 (15.9%)	15 (3.6%)	

Notes: *p*-values represent comparing two different age groups. Gender: Fisher’s Exact; Age: *t*-test; Neophobia level: Pearson’s Chi-squared test; Heard of whole grains: Pearson’s Chi-squared test.

## Data Availability

Data is owned by University of Ljubljana. The original contributions presented in this study are included in the article. Further inquiries can be directed to the corresponding author.

## Supplementary Materials

The following supporting information can be downloaded at: https://www.mdpi.com/article/10.3390/nu18060896/s1. The Supplementary Materials include the complete questionnaire used in the study (File S1), two figures supporting the results, and nine supplementary tables presenting detailed statistical results by age group and food neophobia level: Figure S1: Self-reported preferences for oatmeal preparation methods in school settings by age group; Figure S2: Self-reported preferences for oatmeal preparation methods in school settings by neophobia level; Table S1: Correct identification of whole-grain foods from images, by age group; Table S2: Correct responses to general statements about whole-grain foods, by age group; Table S3: Correct responses to statements about whole-grain bread, by age group; Table S4: Correct responses to statements about whole-grain pasta, by age group; Table S5: Statements reflecting factors for (not) wanting to eat whole-grain foods, and differences in responses by age group and food neophobia level; Table S6: Differences in preferences for including specific grain-based foods in school snacks or lunches, by age group and food neophobia level; Table S7: Differences in preferred preparation methods for oatmeal by age group and food neophobia level; Table S8: Differences in motivational drivers for trying new foods, by age group and food neophobia level; Table S9: Post-hoc comparisons: motivators to try new foods by food neophobia level.

## Abbreviations

The following abbreviations are used in this manuscript:

NIJZ	National Institute of Public Health of Slovenia
GDPR	General Data Protection Regulation
ICFNS	Italian Child Food Neophobia Scale
