# Supplementary material for: Age, Food Neophobia, and Whole-Grain Acceptance in Slovenian Adolescents in the Context of Organized School Meals: Insights from the National “Whole Grain” Project
Source: Nutrients. 2026 Mar 12;18(6):896. doi: 10.3390/nu18060896 (PMC13029692; doi:10.3390/nu18060896)
Supplement: Supplementary file 1 [file nutrients-18-00896-s001.zip › nutrients-4168823-supplementary.pdf]

SUPPLEMENTARY MATERIALS

File S1. Questionnaire on the Acceptability of Whole Grain Products Among Young Consumers, translated from Slovenian language

Q1 - Gender

◯ Female
◯ Male
◯ Other

Q2 - Age (in years). Write the number. ____________________

Q3 – Enrolled in:

◯ Elementary school
◯ Secondary school
◯ Gymnasium

IF (1) Q3 = [2] (Secondary school)
Q4 – Write the name of the program. __________________

Q5 – Class:

◯ 1. class
◯ 2. class
◯ 3. class
◯ 4. class
◯ 5. class (Elementary school)
◯ 6. class (Elementary school)

Q6 - What was your overall grade score last year? Please, mark.

◯ Passable
◯ Good
◯ Very good
◯ Excellent

Q7 - Have you heard of whole grain products before?

◯ Yes
◯ No

Q8 - Consuming whole grain foods and dietary fiber provide several health benefits. Select the benefits that apply below.

Multiple answers possible

☐ They reduce inflammation in the body.
☐ They reduce the risk of gastrointestinal cancer.
☐ They aid in regular digestion.
☐ They cause a rapid spike in blood sugar levels.
☐ They contribute to increased muscle mass.
☐ They contribute to better vision.
☐ They reduce the risk of cardiovascular diseases.

Q9 - Has your school ever offered whole grain options during snack or lunch time? Please mark your answer for both meals.

	Yes	No	I don't know
School snack	◯	◯	◯
School lunch	◯	◯	◯

Q10 - Are the foods shown in the photos whole grain? Please mark the correct response for each item.

		Yes	No	I don't know
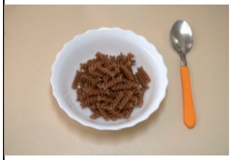	Whole grain pasta	○	○	○
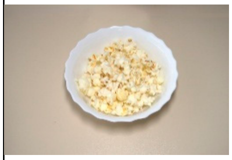	Popcorn	○	○	○
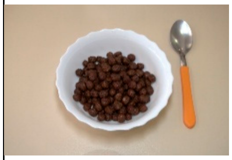	Chocolate breakfast cereals	○	○	○
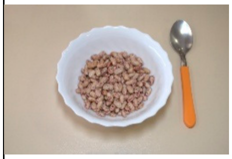	Brown beans	○	○	○
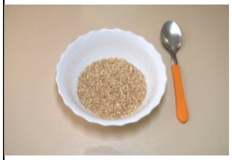	Whole grain rice	○	○	○
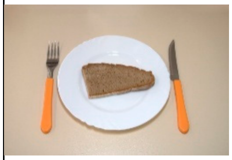	Whole grain bread	○	○	○
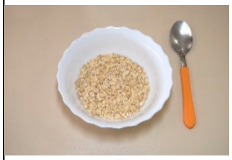	Rolled oats	○	○	○
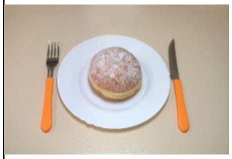	Doughnut with jam	○	○	○
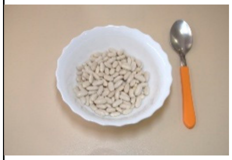	White beans	○	○	○
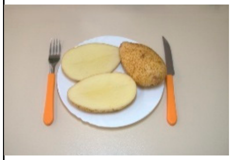	Potato	○	○	○
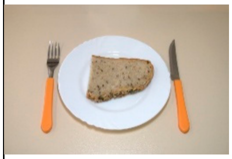	Semi-white bread with seeds	○	○	○

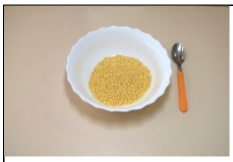	Millet groats	○	○	○
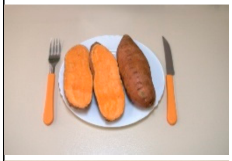	Sweet potato	○	○	○
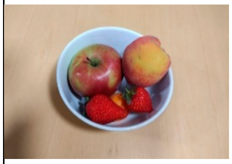	Fruits	○	○	○
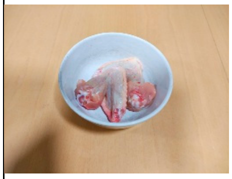	Chicken meat	○	○	○
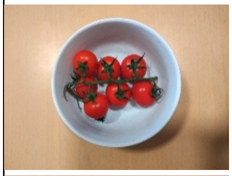	Tomato	○	○	○
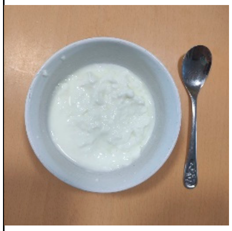	Plain yogurt	○	○	○

Q11 - Are the following statements about whole grain foods true or false? Please mark your answer for every statement.

	True	False	I don't know
A whole wheat kernel consists of germ, the endosperm, and the bran.	○	○	○
Consuming whole grain food is healthy.	○	○	○
It is recommended to store whole grain flour in the refrigerator.	○	○	○
Millet grains can be cooked in milk to prepare a dish called millet milk porridge.	○	○	○
Whole grain bread is more satiating than bread made from white flour.	○	○	○
Whole grain pasta usually takes longer to cook than pasta made from white flour.	○	○	○

Q12 - Are the following statements about whole grain bread true or false? Please mark your answer for every statement.

	True	False	I don't know
Any bread topped with seeds is considered whole grain bread.	○	○	○
Whole grain bread contains more milk than white bread.	○	○	○
Whole grain bread contains the entire ground grain kernel.	○	○	○
Whole grain bread stays fresh longer than white bread.	○	○	○
The crumb of whole grain bread is darker in color than that of white bread.	○	○	○
Whole grain bread contains more vitamins and minerals than white bread.	○	○	○
Whole grain bread is saltier than white bread.	○	○	○

The aroma of whole grain bread differs from that of white bread.			

Q13 - Are the following statements about whole grain pasta true or false? Please mark your answer for every statement.

	True	False	I don't know
Whole grain pasta is the same color as pasta made from white flour.			
The packaging of whole grain pasta always features the label: »Whole grain«.			
Whole grain pasta usually takes longer to cook than pasta made from white flour.			
Whole grain pasta is more satiating than pasta made from white flour.			

Q14 - Do you eat the following foods? Please mark your answer for each one.

	NEVER	RARELY (a few times a year)	SOMETIMES (at least once a month)	OFTEN (several times a week or every day)
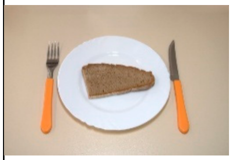 Whole grain bread				
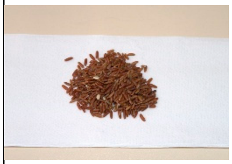 Whole grain/brown rice				
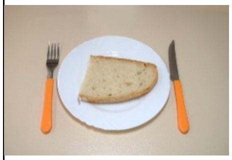 White bread				
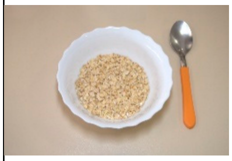 Rolled oats				
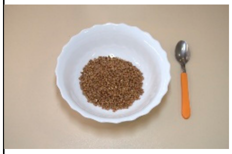 Buckwheat porridge				
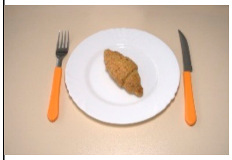 Whole grain croissant				
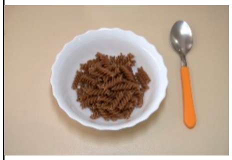 Whole grain pasta				
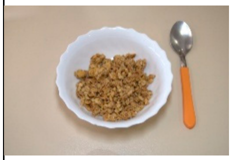 Granola				

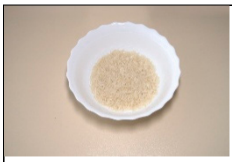	White rice				
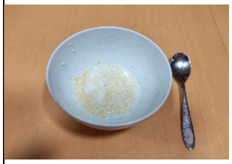	Millet milk porridge				
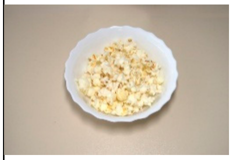	Popcorn				

Q15 - What would encourage you to eat more whole grain foods? Please select all that apply to you. »I would eat whole grain foods more often ...« Please mark a response for each reason listed.

	Strongly disagree	Disagree	Neither agree nor disagree	Agree	Strongly agree
... because it's good for my health.					
… if we ate them more often at home.					
... if I could try just a small portion.					
... if I could prepare and try them myself during class.					
... if they were recommended by people I follow on social media (YT, TikTok, IG, etc.)					
... if they were on the school menu more often.					
... if my classmates and friends ate them.					
... if they were recommended by a famous athlete, musician, or influencer.					
... if it would help me lose some weight.					
... if they tasted sweet, like a dessert.					
... if it would help me build more muscle.					
... if it would help me have a nice body shape.					

Q16 - How often would you like the following foods to be included in your school snack or lunch? Please select an answer for each food item listed.

		NEVER	OCCASIONALLY (once a month)	OFTEN (once a week)	ALWAYS (every day)
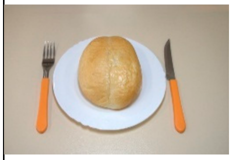	White bread roll				
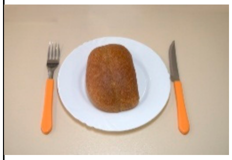	Whole grain bread roll				
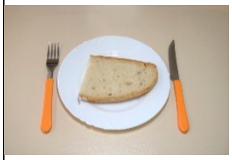	White bread				
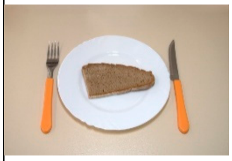	Whole grain bread				

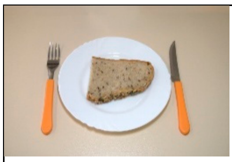	Bread with crushed grains (with seeds)	○	○	○	○
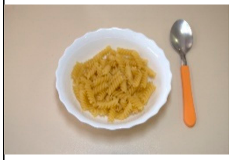	White flour pasta	○	○	○	○
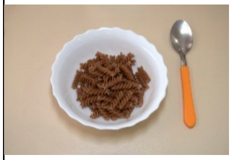	Whole grain pasta	○	○	○	○
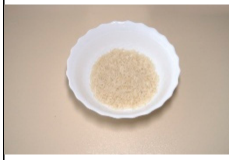	White rice	○	○	○	○
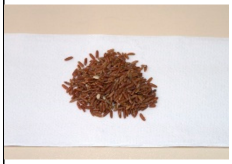	Brown rice	○	○	○	○

Q17 - In your opinion, how should oatmeal be prepared for school snacks to make it taste good so that you would eat it? »It should be prepared with...« Please mark a response for each preparation method.

	NOT AT ALL (I wouldn't eat it)	MAYBE (I would eat it)	VERY LIKELY (I would eat it)	OF COURSE (I would eat it)
... milk.	○	○	○	○
... plain yogurt.	○	○	○	○
... plant-based drink (soy, oat, almond).	○	○	○	○
... fruit yogurt.	○	○	○	○
... milk and dried fruit.	○	○	○	○
... chocolate pieces, milk, and dried fruit.	○	○	○	○
... milk and nuts.	○	○	○	○
... milk and cocoa powder.	○	○	○	○

Q18 - Write your own suggestion for how to prepare oatmeal (optional question). ___________________

Q19 - What makes you try a new food and keep eating it? »I'll try an unfamiliar dish and start eating it regularly if ...« Please select an answer for every reason.

	Not at all	Maybe	Very likely	Of course
... it tastes good when I try it for the first time.	○	○	○	○
... I've seen the dish on TikTok, YT, IG, etc.	○	○	○	○
... my friend also likes this dish.	○	○	○	○
... the dish has a nice color.	○	○	○	○
... the dish is healthy.	○	○	○	○
... we talked about it in class.	○	○	○	○
... the new dish is served with tasty side dishes and foods.	○	○	○	○
... we talked about the new dish at home.	○	○	○	○
... it's also served at my favorite restaurant.	○	○	○	○
... the dish has a sweet taste.	○	○	○	○
... I were very hungry.	○	○	○	○
... if I would get a double dessert as a reward at lunch.	○	○	○	○

Q20 - Write your own reason for trying and continuing to eat a new dish (optional). ___________________

Q21 - Please indicate how much you agree with the following statement about eating food.

	Strongly disagree	Disagree	Neither agree nor disagree	Agree	Strongly agree
I eat new and unusual foods (dishes) almost every day	◯	◯	◯	◯	◯
I like trying different flavors and unusual foods (dishes) from various countries.	◯	◯	◯	◯	◯
When I am at a friend's party, I like to try new foods (dishes).	◯	◯	◯	◯	◯
I don't trust new foods (dishes).	◯	◯	◯	◯	◯
If a food (dish) is new, I don't try it.	◯	◯	◯	◯	◯
I am afraid to try foods (dishes) that I have never had before.	◯	◯	◯	◯	◯
I am a very picky eater.	◯	◯	◯	◯	◯
I eat everything—absolutely everything!	◯	◯	◯	◯	◯

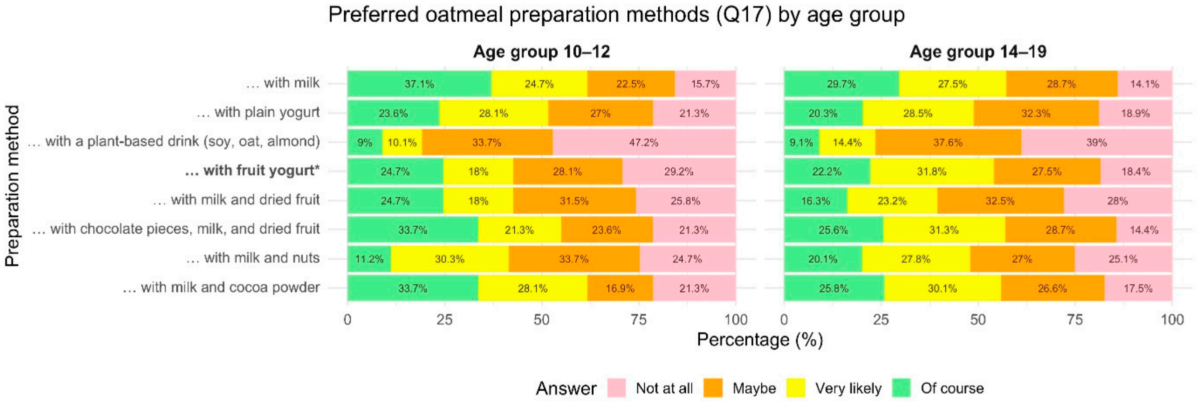

Figure S1. Self-reported preferences for oatmeal preparation methods in school settings by age group (10–12 vs 14–19 years). Stacked bar chart showing the percentage distribution of responses across four likelihood categories (not at all to of course) for each preparation method. Statistical differences between neophobia groups were assessed using Fisher's exact test. Preparation methods marked with * differ significantly between groups ($p < 0.05$).

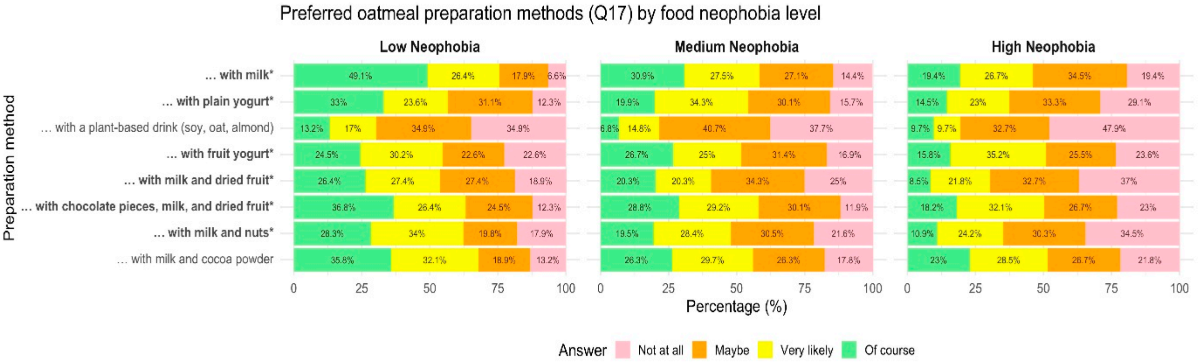

Figure S2. Self-reported preferences for oatmeal preparation methods in school settings by neophobia level (low vs. medium vs. high). Stacked bar chart showing the percentage distribution of responses across four likelihood categories (not at all to of course) for each preparation method. Statistical differences between age groups were assessed using Fisher's exact test. Preparation methods marked with * differ significantly between groups ($p < 0.05$).

Table S1. Correct identification of whole grain foods (in italic) from images, by age group. This table presents the percentage and number of participants (% (n)) in each age group (10–12 and 14–19) who correctly identified whether the food item shown in the photograph is whole grain.

**Food item**	**Total % (n=507)**	**Age 9–12 % (n=89)**	**Age 14–19 % (n=418)**	**p-value***
Whole grain pasta	95.1 (482)	95.5 (85)	95.0 (397)	1.00
Popcorn	11.0 (56)	10.1 (9)	11.2 (47)	0.90
Chocolate cereal balls	83.2 (422)	78.7 (70)	84.2 (352)	0.26
Brown beans	61.3 (311)	67.4 (60)	60.0 (251)	0.24
Whole grain rice	95.5 (484)	93.3 (83)	95.9 (401)	0.41
Whole grain bread	96.8 (491)	96.6 (86)	96.6 (405)	1.00
Oat flakes	44.8 (227)	49.4 (44)	43.8 (183)	0.39
Jam-filled doughnut	88.8 (450)	83.1 (74)	90.0 (376)	0.09
White beans	70.4 (357)	70.8 (63)	70.3 (294)	1.00
Potatoes	84.4 (428)	86.5 (77)	84.0 (351)	0.66
Semi-white bread with seeds	34.9 (177)	21.3 (19)	37.8 (158)	**0.005***
Millet groats	37.9 (192)	53.9 (48)	34.4 (144)	**0.001***
Sweet potatoes	84.8 (430)	85.4 (76)	84.7 (354)	0.99
Fruit	89.5 (454)	87.6 (78)	90.0 (376)	0.65
Chicken meat	91.9 (466)	91.0 (81)	92.1 (385)	0.89
Tomato	89.7 (455)	84.3 (75)	90.9 (380)	0.09
Plain yogurt	89.0 (451)	84.3 (75)	90.0 (376)	0.17

*Statistically significant differences between age groups are indicated by p-values from Pearson's Chi-squared tests.

Table S2. Correct responses to general statements about whole grain foods, by age group. The table shows the percentage and number of participants (% (n)) who correctly evaluated statements related to whole grain food characteristics.

**General statements about whole grain foods**	**Total % (n=507)**	**Age 10–12 % (n=89)**	**Age 14–19 % (n=418)**	**p-value***
A whole wheat kernel consists of germ, the endosperm, and the bran.	66.3 (336)	37.1 (33)	72.5 (303)	**< 0.001***
Consuming whole grain food is healthy.	90.7 (460)	87.6 (78)	91.4 (382)	0.36
It is recommended to store whole grain flour in the refrigerator.	78.5 (398)	83.1 (74)	77.5 (324)	0.30
Millet groats can be cooked in milk to prepare a dish called millet milk porridge.	66.9 (339)	60.7 (54)	68.2 (285)	0.21
Whole grain bread is more satiating than bread made from white flour.	62.9 (319)	64.0 (57)	62.7 (262)	0.90
Whole grain pasta usually takes longer to cook than pasta made from white flour.	51.3 (260)	33.7 (30)	55.0 (230)	**< 0.001***

*Statistically significant differences between age groups are indicated by p-values from Pearson's Chi-squared tests.

Table S3. Correct responses to statements about whole grain bread, by age group. The table shows the percentage and number of participants (% (n)) who correctly evaluated statements related to whole grain bread.

**Statements about whole grain bread**	**Total % (n=507)**	**Age 10–12 % (n=89)**	**Age 14–19 % (n=418)**	**p-value***
Any bread topped with seeds is considered whole grain bread.	80.5 (408)	68.5 (61)	83.0 (347)	**0.003***
Whole grain bread contains more milk than white bread.	58.4 (296)	56.2 (50)	58.9 (246)	0.73
Whole grain bread contains the entire ground grain kernel.	63.3 (321)	59.6 (53)	64.1 (268)	0.49
Whole grain bread stays fresh longer than white bread.	46.4 (235)	56.2 (50)	44.3 (185)	**0.050**
The crumb of whole grain bread is darker in color than that of white bread.	87.4 (443)	84.3 (75)	88.0 (368)	0.43
Whole grain bread contains more vitamins and minerals than white bread.	79.7 (404)	68.5 (61)	82.1 (343)	**0.006***
Whole grain bread is saltier than white bread.	55.0 (279)	48.3 (43)	56.5 (236)	0.19
The aroma of whole grain bread differs from that of white bread.	73.0 (370)	68.5 (61)	73.9 (309)	0.36

*Statistically significant differences between age groups are indicated by p-values from Pearson's Chi-squared tests.

Table S4. Correct responses to statements about whole grain pasta, by age group. The table presents the proportion and number of participants who correctly evaluated claims related to whole grain pasta, split by age group.

**Statements about whole grain pasta**	**Total % (n=507)**	**Age 10–12 % (n=89)**	**Age 14–19 % (n=418)**	**p-value***
Whole grain pasta is the same color as pasta made from white flour.	88.4 (448)	79.8 (71)	90.2 (377)	**0.009***
The packaging of whole grain pasta always features the label: »Whole grain«.	71.4 (362)	76.4 (68)	70.3 (294)	0.31
Whole grain pasta usually takes longer to cook than pasta made from white flour.	49.3 (250)	38.2 (34)	51.7 (216)	**0.030***
Whole grain pasta is more satiating than pasta made from white flour.	64.1 (325)	57.3 (51)	65.6 (274)	0.18

*Statistically significant differences between age groups are indicated by p-values from Pearson's Chi-squared tests.

Table S5. Statements reflecting factors for (not) wanting to eat whole grain foods, and differences in responses by age group and food neophobia level. Responses were rated on a 5-point scale from strongly disagree to strongly agree. Reported p-values indicate whether responses to each statement differed significantly between age groups (10–12 vs. 14–19 years) or between groups with low, medium and high food neophobia.

**Statements about whole grain foods**	**Age effect p-value**	**Neophobia effect p-value**
... because it's good for my health.	**<0.001***	**<0.001***
… if we ate them more often at home.	**0.047***	0.09
... if I could try just a small portion.	0.12	0.05
... if I could prepare and try them myself during class.	**0.026***	**0.014***
... if they were recommended by people I follow on social media (YT, TikTok, IG, etc.)	**<0.001***	0.15
... if they were on the school menu more often.	**0.017***	**0.001***
... if my classmates and friends ate them.	0.17	0.11
... if they were recommended by a famous athlete, musician, or influencer.	**0.003***	**<0.001***
... if it would help me lose some weight.	0.08	0.68
... if they tasted sweet, like a dessert.	**0.006***	0.11

... if it would help me build more muscle.	**0.001***	0.71
... if it would help me have a nice body shape.	**0.009***	0.82

*Statistically significant differences between age groups are indicated by p-values from Fisher's exact tests.

Table S6. Differences in preferences for including specific grain-based foods in school snacks or lunches, by age group and food neophobia level. Participants indicated how often they would like each food to be included, using a 4-point scale from never to always. Reported p-values indicate whether preferences differed significantly between age groups (10–12 vs. 14–19 years) or between groups with low, medium and high food neophobia.

Specific grain-based foods	Age effect p-value	Neophobia effect p-value
Bread with crushed grains (with seeds)	**0.032***	**0.001***
Brown rice	0.09	**0.021***
White flour pasta	0.10	0.74
White bread	0.66	0.06
White bread roll	**<0.001***	0.69
White rice	**0.049***	0.44
Whole grain bread	0.49	**<0.001***
Whole grain bread roll	0.33	0.19
Whole grain pasta	**0.020***	0.38

*Statistically significant differences between age groups are indicated by p-values from Fisher's exact tests.

Table S7. Differences in preferred preparation methods for oatmeal by age group and food neophobia level. Participants indicated how likely they would be to taste and eat oatmeal at school depending on how it was prepared, using a 4-point scale from not at all to of course. Reported p-values reflect whether preferences differed significantly between age groups (10–12 vs. 14–19 years) or between groups with low, medium and high food neophobia.

Statements about whole grain foods	Age effect p-value	Neophobia effect p-value
... with a plant-based drink (soy, oat, almond)	0.49	0.07
... with chocolate pieces, milk, and dried fruit	0.06	**0.006***
... with fruit yogurt	**0.024***	**0.031***
... with milk and cocoa powder	0.15	0.20
... with milk and dried fruit	0.28	**0.002***
... with milk and nuts	0.20	**0.002***
... with milk	0.45	**<0.001***
... with plain yogurt	0.72	**0.001***

*Statistically significant differences between age groups are indicated by p-values from Fisher's exact tests.

Table S8. Differences in Motivational drivers for trying new foods by age group and food neophobia level. Participants indicated what would motivate them to try new foods, using a 4-point scale from not at all to of course. Reported p-values reflect whether motivations differed significantly between age groups (10–12 vs. 14–19 years) or between groups with low, medium and high food neophobia.

Motivational Driver	Age effect p-value	Neophobia effect p-value
... if I were very hungry	**0.006***	**<0.001***
... if I would get double dessert as a reward at lunch	**<0.001***	0.08
... if I've seen the dish on TikTok, YouTube, Instagram, etc.	**<0.001***	**0.024***
... if it tastes good when I try it for the first time	0.23	**<0.001***
... if it's also served in my favorite restaurant	**0.001***	**0.001***
... if my friend also likes this dish	0.43	**0.003***
... if the dish has a nice color	**<0.001***	0.29
... if the dish has a sweet taste	**0.003***	0.94
... if the dish is healthy	**0.042***	**<0.001***
... if the new dish is served with tasty side dishes and foods	0.31	**<0.001***
... if we talked about it in class	0.36	**0.012***
.. if we talked about the new dish at home	0.44	**<0.001***

*Statistically significant differences between age groups are indicated by p-values from Fisher's exact tests.

Table S9. Post-hoc comparisons: motivators to try new foods by food neophobia level (low vs. medium vs. high).

Motivational Factor	Comparison	Adjusted p-value
…if I were very hungry	Low vs. High	< 0.001
…if I were very hungry	Medium vs. High	0.001
…if I were very hungry	Low vs. Medium	0.025
…if the dish is healthy	Low vs. High	< 0.001
…if the dish is healthy	Medium vs. High	0.001
…if it tastes good when i try it for the first time	Low vs. High	< 0.001
…if it tastes good when i try it for the first time	Low vs. Medium	0.001
…if the new dish is served with tasty side dishes and foods	Low vs. High	< 0.001
…if we talked about the new dish at home	Low vs. High	< 0.001
…if we talked about the new dish at home	Medium vs. High	< 0.001
…if we talked about the new dish at home	Low vs. Medium	0.009
…if we talked about it in class	Low vs. High	0.003
…if it's also served in my favorite restaurant	Low vs. High	0.002
…if it's also served in my favorite restaurant	Medium vs. High	0.002
…if my friend also likes this dish	Medium vs. High	0.01
